# IL-1β enhances cell viability and decreases 5-FU sensitivity in novel colon cancer cell lines derived from African American patients

**DOI:** 10.3389/fonc.2022.1010380

**Published:** 2022-12-01

**Authors:** Marzia Spagnardi, Jenny Paredes, Jovanny Zabaleta, Jone Garai, Tiana Reyes, Laura A. Martello, Jennie L. Williams

**Affiliations:** ^1^ Department of Medicine, Division of Gastroenterology and Hepatology, SUNY Downstate Health Sciences University, Brooklyn, NY, United States; ^2^ Department of Interdisciplinary Oncology, Louisiana State University Health Sciences Center, New Orleans, LA, United States; ^3^ Stanley S. Scott Cancer Center, Louisiana State University Health Sciences Center, New Orleans, LA, United States; ^4^ Department of Family, Population and Preventive Medicine, Stony Brook, Stony Brook University, NY, United States

**Keywords:** African American, colon cancer, health disparities, Interleukin-1β, 5-FU, IL-1Ra

## Abstract

**Background:**

In the U.S., African Americans (AAs) present with the highest incidence and mortality rates for Colorectal Cancer (CRC). When compared to Caucasian American (CA) patients, AAs also have reduced response to the first line standard of care chemotherapeutic agent 5-Fluorouracil (5-FU). Previously, we observed differential gene expression between the two populations, suggesting that colon tumors from AA patients display a decreased antitumor immune response and an increased expression of genes encoding proteins involved in inflammatory processes, such as Interleukin-1β (IL-1β). Here, we investigate the role of IL-1β in modifying chemotherapeutic response and altering expression of proteins in novel AA and well-established CA colon cancer cell lines.

**Methods:**

RNA sequencing analysis was performed to detect expression of genes involved in inflammation in AA and CA colon cancer cells. The effects of IL-1β on 5-FU response was evaluated by assessing cell viability (MTS assay) and apoptosis (flow cytometry analysis) following treatment with 5-FU alone or in combination with the cytokine. Further, we used an IL-1 receptor antagonist (IL-1Ra) to inhibit IL-1β-induced effects on 5-FU sensitivity and NF-kB pathway activation.

**Results:**

AA colon cancer cell lines present significant increase in expression of genes *IL1R2* (373-fold change (FC), *IRAK1 (*3.24 FC), *IKBKB*, (5.33 FC) *NF-KB IA* (5.95 FC), *MYD88*, (3.72 FC), *IRAK3* (161 FC), *TRAF5* (4.1 FC). A significant decrease in the response to 5-FU treatment, as well as a significant increase in phosphorylation of IκBα and secretion of IL-8, was seen following IL-1β treatment, in both AA and CA cell lines. Finally, treatment with IL-1Ra was able to reverse the effects induced by IL-1β, by increasing the cells sensitivity to 5-FU. IL-1Ra also inhibited phosphorylation of IκBα and IL-8 secretion.

**Conclusions:**

Our results suggest a differential expression of inflammatory genes and proteins that might regulate the different response to IL-1β between AA and CA colon cancer cell lines. Our data also demonstrates that IL-1β is involved in modulating 5-FU response in both AA and CA colon cancer cell lines. Further investigation of these mechanisms might help elucidate the differences seen in incidence, mortality and response to therapy in AA colon cancer patients.

## Introduction

Colorectal cancer (CRC) is the third most common cancer and the third cause of cancer-related deaths in the US (1). Prognosis of CRC depends on stage at diagnosis, location and molecular characteristics of the tumors, which can be classified based on their microsatellite status as microsatellite stable (MSS), which presents a worse prognosis, and microsatellite instable (MSI), which offers a better prognosis but worse response to adjuvant chemotherapy (2, 3). When looking at different races and ethnicities, African Americans (AA) present the highest risk for CRC and they are more likely to die from this disease than Caucasian Americans (CA) (4). Despite the efforts to close the risk and mortality gaps between AA and CA colorectal cancer patients, the information available to us concerning chemotherapeutic responses and molecular alterations are solely based on population studies (4–8). The lack of commercially available *in vitro* models of AA origins represents a critical barrier in understanding CRC mechanisms in this population. Although reasons for the disparities might be related to socioeconomic status, with lower rates of screening and lack of access to optimal treatment options (9), studies have shown that AA tend to have a decreased response to 5-Fluoroacil (5-FU) based therapy (4) and they also appear to have lower frequency of MSI tumors (5), which have been shown to be more likely to respond to immunotherapy (10). Recent findings have demonstrated the presence of genes uniquely mutated in AA colorectal cancer patients (7), as well as genes differentially expressed, or differentially methylated, that are involved in anti-tumoral immunity and inflammatory pathways (7, 8).

In agreement with these findings, transcriptomic analysis from our lab of tumor/non-tumor samples collected from AA and CA patients found that tumors from AAs present a lower expression of *Granzyme B* (*GZMB*) and an impaired infiltration of cytotoxic CD8+ T cells, suggesting an overall decreased antitumoral activity in AAs when compared to CAs (11). Furthermore, our data shows an increased expression of genes encoding for pro-inflammatory cytokines IL-1β and IL-8 in tumors from AA patients, suggesting a possible correlation between inflammation and disease progression in this population (11). Inflammation has been identified as a risk factor and a contributor in developing different types of cancer, including CRC (12). Clinical studies have demonstrated that CRC patients present altered cytokine production, both systemically and locally (13). Moreover, it has been seen that cytokines can act as cancer cell survival factors and promote tumor growth and angiogenesis, as well as suppress immune-mediated tumor elimination (12, 14). Specifically, IL-1β has been previously associated with promoting CRC (12), and findings have shown a role for IL-1β in interacting with immune cells and supporting tumor progression (15) as well as invasiveness and resistance to certain chemotherapeutic agents (16).

In this study we utilized two novel African American colon cancer cell lines, generated by Dr. Jennie Williams’ laboratory (17), to evaluate the effects induced by IL-1β and to compare the results in established CA colon cancer cell lines. The aforementioned cell lines have been previously characterized in terms of microsatellite status, expression of proteins associated with CRC (p53, β-catenin, Msh2, Msh6, Mlh1) and ability to generate tumors in mouse models (17). These cells will be used here to determine the mechanisms of cell growth and drug response in the context of inflammation and, more specifically, the IL-1β pathway. We plan to investigate the role of IL-1β in promoting colon cancer cells proliferation, the effects of IL-1β on 5-FU response and the molecular mechanisms involved in the effects induced by IL-1β.

## Materials and methods

### Cell culture and reagents

CA colon cancer cell lines HT-29 (MSS) and HCT 116 (MSI) were purchased from the American Type Culture Collection (Manassas, VA), while AA colon cancer cell lines, CHTN-06 (MSS) and SB-521 (MSI), were generated at Stony Brook University as previously described (17). All cell lines were maintained in DMEM media (Corning) supplemented with 10% FBS, 100 U/ml penicillin, 100 U/ml streptomycin, 4.5 g/L glucose and sodium pyruvate, in humidified incubator with 37°C and 5% CO_2_. Human recombinant IL-1β was purchased from Cell Signaling Technology and resuspended in PBS for a final stock concentration of 10 ug/ml (catalog #8900). 5-FU was purchased from Sigma-Aldrich (catalog #F6627-1G) and resuspended in DMSO (Thermo Fisher Scientific) for a final stock concentration of 380mM. Human recombinant IL-1Ra was purchased from Sigma-Aldrich (catalog ##SRP3084) and resuspended in PBS supplemented with 0.1% BSA for a final stock concentration of 100 ug/ml.

### RNA sequencing

RNA sequencing and analysis was done at the Translational Genomics Core (TGC) at the Stanley S. Scott Cancer Center, LSUHSC, New Orleans, LA, as we have done previously (11). Briefly, RNA was isolated from untreated cells using the Universal RNA/DNA Isolation kit (Qiagen) according to manufacturer’s protocol. Isolated RNA was quantified by Qubit (ThermoFisher) and checked for RNA integrity using the Agilent BioAnalyzer 2100 (Agilent). Paired-end libraries (2 x 75) were prepared using the TruSeq Stranded mRNA Library Prep kit, validated, and normalized following the recommendations of the manufacturer (Illumina). Libraries were sequenced in the NextSeq500 using a High Output Kit v2.5, 150 cycles from Illumina. FASTQ output files were uploaded to Partek Flow, contaminants (rDNA, tRNA, mtrDNA) were removed using Bowtie2 (version 2.2.5) and the unaligned reads were then aligned to STAR (version 2.5.3a). Aligned reads were quantified to the hg19- RefSeq Transcripts 93 normalized by log2 (x+1) transformation. Normalized counts were used to determine differential gene expression between AA cell lines and CA cell lines.

### Cell viability assays

3-(4,5-dimethylthiazol-2-yl)-5-(3-carboxymethoxyphenyl)-2-(4-sulfophenyl)-2H-tetrazolium (MTS) assays were performed using the CellTiter 96 AQueous One Solution Cell Proliferation Assay (G3582; Promega) according to the manufacturer’s instructions. For IL-1β studies, cells were seeded in triplicate in 96-well plates at 5 x 10^3^ cells/well in complete DMEM in presence of different concentrations of IL-1β and incubated overnight at 37°C in 5% CO2. Concentrations were determined based on previous studies involving IL-1β and colon cancer cell lines (18, 19). The MTS reagent (20 ul/well) was added 24, 48, 72 and 96 hours after seeding and cells were incubated at 37°C for 2.5 hours. Cell viability was measured by absorbance (490 nm) using a plate reader BioTek Lx800. For the inhibition studies with IL-1Ra, cells were seeded as previously described, in the presence of IL-1β (10 ng/ml). Twenty-four hours after seeding, cells were treated with IL-1Ra (10 ug/ml) and incubated at 37°C for 24, 48 and 72 hours. For 5-FU cytotoxicity assay, cells were seeded as previously described, in complete DMEM alone or containing 10 ng/ml of IL-1β for 24 hours and then treated with different concentration of 5-FU (1, 2.5, 5, 10, 15 uM) for 72 hours.

### Enzyme-linked immunosorbent assays

Secretion of IL-8 was measured in the culture media of the cell lines using the RayBio^®^ Human IL-8 (CXCL8) ELISA Kit according to manufacturer’s instructions (catalog #ELH-IL8-1). A group of 2.5 x 10^4^ cells were seeded in triplicate in 12-well plates in DMEM supplemented with 10% FBS, in the presence of IL-1β (10 ng/ml), and incubated overnight. Twenty-four hours later, cells were treated with IL-1Ra (10 ug/ml) for 24 hours. Cell supernatants were collected and stored at -20°C until use.

### Western blot

For IL-1 Receptor detection, 5.0 x 10^5^ untreated cells were seeded in 10cm plates in DMEM supplemented with 10% FBS and incubated for 48 hours. The cells were then harvested, washed with PBS and membrane and cytoplasmic fractions were obtained by using the MEM-PER Plus Kit (Thermo Fisher #89842), supplemented with Protease and Phosphatase Inhibitors (ThermoFisher #78425). For detecting the effects of IL-1β on protein phosphorylation, 3.0 x 10^5^ cells were seeded in 6 well plates and incubated overnight. The following day, cells were serum starved (serum free media) for 2 hours and then treated with 10 ng/ml of IL-1β for 15 minutes. At the end of treatment period, the cells were washed with PBS and lysed on ice with RIPA lysis buffer supplemented with Protease and Phosphatase Inhibitors (ThermoFisher #78425). Protein concentration was determined by the DC Protein assay (Bio-Rad Laboratories #5000113, #5000114, #5000115) and absorbance measured using the BioTek ELx800 Microplate Reader. Isolated proteins were separated by gel electrophoresis using 12% precast polyacrylamide gels (Bio-Rad Laboratories, catalog ##4561044) and transferred onto polyvinylidene difluoride (PVDF) membranes (Bio-Rad Laboratories, catalog #1620175). Probing with primary antibodies IL-1R H8 (Santa Cruz, 1:500, catalog #sc-393998), Na,K-ATPase (Cell Signaling Technology, 1:1000, #3010), phospho-IkBα (Cell Signaling Technology 1:500, catalog #2859), total IkBα (Cell Signaling Technology 1:1000, catalog #4812), and α-tubulin (Cell Signaling Technology, 1:1000, catalog #2125) was performed overnight at 4°C. Finally, blots were incubated with secondary antibodies mouse anti-goat (Santa Cruz, catalog# sc-544183) and anti-rabbit IgG, HRP-linked (Cell Signaling Technology, catalog #7074), for 1 h at room temperature. Protein expression was detected using an enhanced chemiluminescence reaction kit (ThermoFisher #34580). Images of the blots were analyzed using ImageJ analysis program and all proteins were compared to the loading control α-tubulin.

### Flow cytometry

1.5 x 10^5^ cells were seeded in 6-well plates in DMEM supplemented with 10% FBS, in presence or absence of IL-1β (10 ng/ml), and incubated overnight. The following day, cells were treated with either DMSO, or 5-FU (2.5 or 5 µM) and incubated for 72 hours. Cells were harvested and washed with PBS two times. The samples were then resuspended in FACS binding buffer (5% FBS in 1X PBS) and incubated for 30 minutes. The cells were then stained with Annexin V, Propidium Iodide (PI), or both, for 45 minutes. The population of viable cells and apoptotic cells was evaluated by flow cytometry, using Novocyte 3000 Flow Cytometer, and analyzed by Novoexpress Software.

### Statistical analyses

Statistical analysis was performed using the GraphPad Prism 9.0 software. Experiments were repeated three times, either in duplicates or triplicates. Data are presented as mean ± SEM. Two-tailed paired student t-test was applied to compare difference in two groups, p < 0.05 was considered as statistically significant.

## Results

### AA and CA colon cancer cell lines show differentially expressed genes involved in IL-1, MAPK and NF-κB pathways

**Figure 1:**
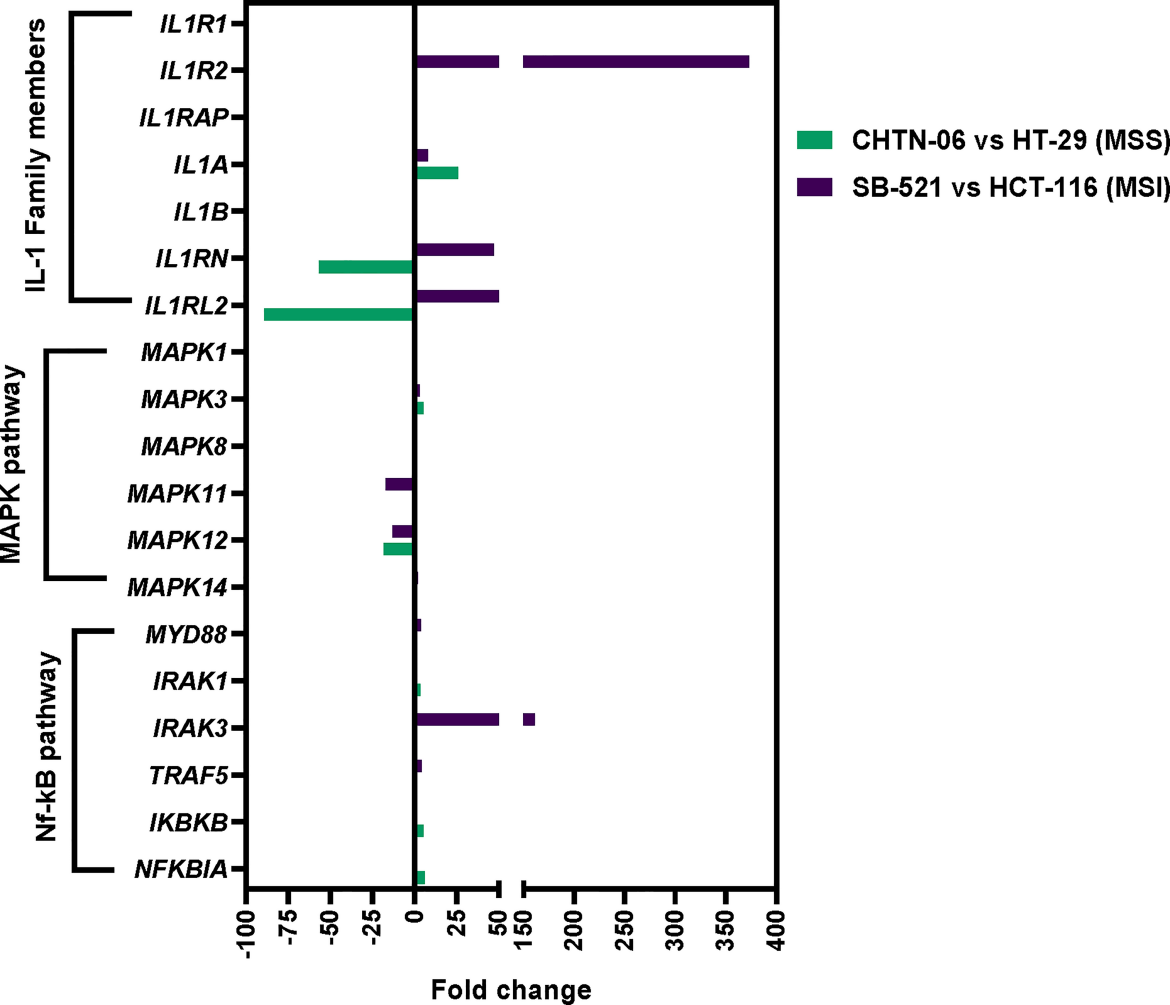
Differential gene expression of *ILB*, *MAPK*, and *NFKB* related genes between AA and CA colon cancer cell lines. Cell lines have been grouped based on their microsatellite status in MSS (CHTN-06 vs. HT-29) and MSI (SB-521 vs. HCT-116). Graphical representation of fold change of genes compared between cell lines.

To better understand the differences in response to the IL-1β cytokine observed between the AA and CA colon cancer cell lines, we performed RNA sequencing analysis and focused on the expression of the genes involved in the IL-1β pathway. Specifically assessed were the IL-1 Receptor family genes, the MAPKinase genes, and the NF-κB pathway genes (**Figure 1**). According to the analysis, several genes from the aforementioned pathways were upregulated in AA colon cancer cell lines compared to CA cell lines, with the most striking differences seen in the MSI cell lines. Among the three IL-1 Receptor genes, we found *IL-1 Receptor Type 2* (*IL1R2*) to be upregulated in SB-521, with a 373-fold increase, while there was no significant difference in the expression of both *IL-1 Receptor Type 1 (IL1R1)* and *IL-1 Receptor Accessory Protein (IL1RAP)* in either comparison. An increased expression was also seen for *Interleukin-1-alpha* (*IL1A*) gene for both CHTN-06 and SB-521 (25.7- and 7.67-fold change, respectively), as well as an increase in the expression of *IL-1 Receptor antagonist* (*IL1RN*) in SB-521 (47.9-fold change) and a decrease in CHTN-06 (-56.7-fold change). We did not see any significant differences in the expression of *Interleukin-1-beta* (*IL1B*) between cell lines. Based on these results, we tested the cell lines for their baseline secretion of these altered cytokines: no secretion was found for either IL-1A, IL-1β and IL-1Ra for any of the cell lines, suggesting that the differences seen at the transcript level were not reflected at the protein level (Data not shown). For the MAPKinase gene family, the *Mitogen-Activated Protein Kinase 3* (*MAPK3*) was found upregulated in both AA cell lines, while the *Mitogen-Activated Protein Kinase 12* (*MAPK12*) was downregulated in these same cell lines. Specific to the MSI cell line SB-521, we found that *Mitogen-Activated Protein Kinase 11* and *Mitogen-Activated Protein Kinase 14* (*MAPK11* and *MAPK14*) were downregulated and upregulated, respectively, when compared to HCT-116. Most interesting, the analysis demonstrated that multiple genes involved in the NF-κB pathway were upregulated in both of the AA MSS and MSI cell lines. For example, we found that *Interleukin 1 Receptor Associated Kinase 1* (*IRAK1*, 3.24 fold-change), *Inhibitor Of Nuclear Factor Kappa B Kinase Subunit Beta* (*IKBKB*, 5.33-fold change) and *NF-KB Inhibitor Alpha* (*NF-KB IA*, 5.95-fold change) were upregulated in CHTN-06 (MSS). Whereas *Myeloid differentiation primary response 88* (*MYD88*, 3.72-fold change), *Interleukin 1 Receptor Associated Kinase 3* (*IRAK3*, 161-fold change), and *TNF Receptor Associated Factor 5* (*TRAF5*, 4.1-fold change) were upregulated in SB-521 (MSI). These findings suggest a differential expression of specific genes involved in pro-inflammatory processes, which appears to be independent of the MSS or MSI status of the cell lines.

**Figure 2:**
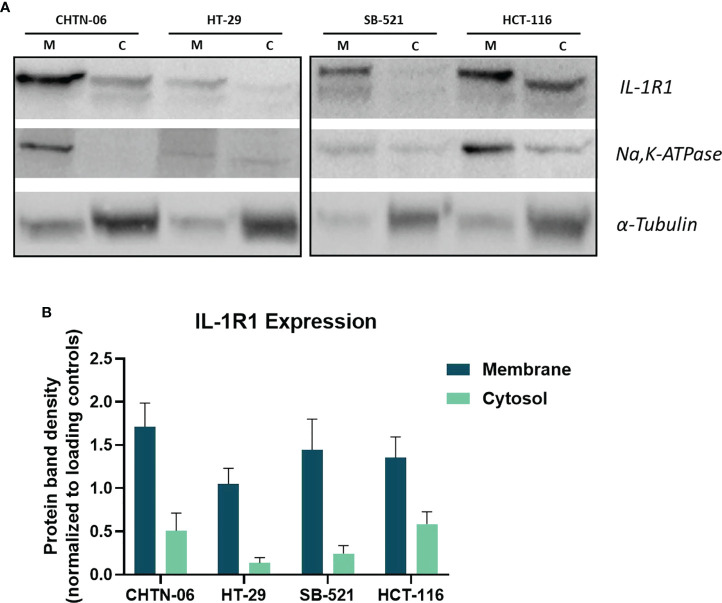
Expression levels IL-1R1 in membrane and cytosol fractions in AA and CA colon cancer cell lines. **(A)** Representative images of Western Blot for detection of IL-1R1. Na,K-ATPase and α-tubulin were used as loading controls. M= Membrane, C= Cytosol. **(B)** Densitometry analysis of IL-1R1 levels in membrane and cytosol fractions. Membrane protein was normalized to ATPase and cytosolic protein was normalized to α-tubulin. Data are representative of three independent experiments. Error bars represent SEM.

Since our interest is to investigate the effects induced by IL-1, and since the main signaling transducing receptor for this cytokine is IL-1R1, we evaluated the membrane expression of this protein. For this experiment, membrane and cytoplasmic fractions were isolated and the expression levels of IL-1R1 were evaluated in both fractions *via* western blot analysis. To provide quality control for the membrane and cytosol separation procedure, Na, K-ATPase and a-tubulin were used as loading controls for membrane and cytosol fractions, respectively. As **Figure 2** illustrates, the receptor is expressed on the membrane of all four cell lines. We found that there were no statistically significant differences between AA CHTN-06 (mean 1.71) and CA HT-29 (mean 1.08) cell lines, or between AA SB-521 (mean 1.44) and CA HCT-116 (mean 1.35) cell lines.

### IL-1β decreases sensitivity to 5-FU treatment in colon cancer cell lines

To assess whether IL-1β promotes cell proliferation in the AA colon cancer cell lines and how this response may differ from that seen in CA cell lines, we performed an MTS assay and tested different concentrations of IL-1β for multiple time points. The IL-1β concentrations were derived from previous literature, with concentrations ranging from 1.25 to 160 ng/ml (18, 19). Our results show a significant concentration-dependent increase in cell proliferation when the cells were treated with IL-1β after 96 hours (**Supplemental Figure 1**). Interestingly, AA MSS cell lines CHTN-06 and SB-521 significantly increases cell proliferation after treatment with 1, 5, 10, 30 and 60 ng/ml of IL-1β, while for the CA cell lines, we only saw a significant increase starting at 10ng/ml (HT-29) or 5ng/ml (HCT-116) of treatment. These differences in response are constant throughout different time points tested (24, 48, 72 hours. Data not shown). Our data also shows that the highest increase (fold change) in cell proliferation was seen when the cells were treated with 10 ng/ml, reaching a plateau point, therefore we proceeded to use 10 ng/ml as IL-1β concentration for subsequent experiments.

**Figure 3:**
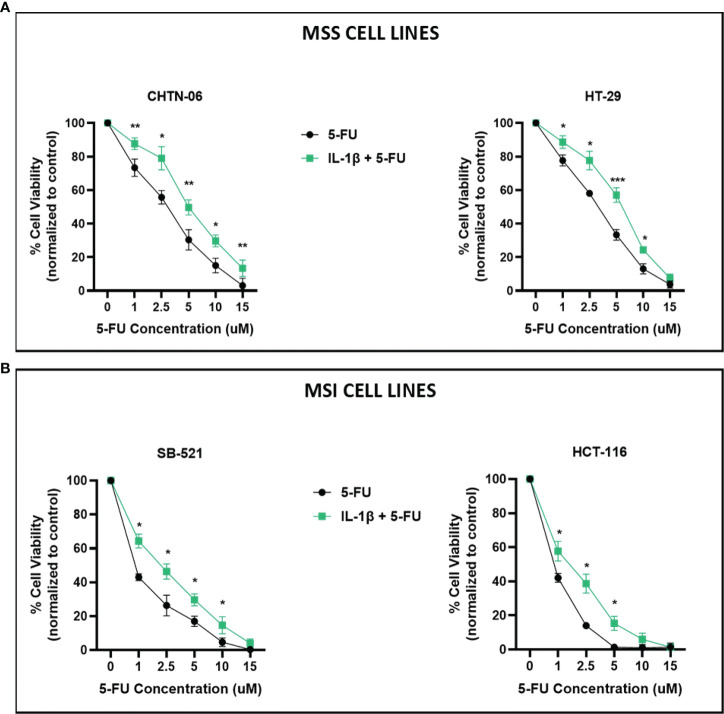
IL-1β decreases sensitivity to 5-FU treatment. Cell viability following treatment with 5-FU, in the presence or absence of IL-1β, was determined by MTS assay. Cells were seeded in media containing 10 ng/ml of IL-1β and then treated with increasing concentrations of 5-FU and incubated for 72 hours. **(A)** CHTN-06 AA cell lines, MSS; HT-29 CA cell line, MSS; **(B)** SB-521 AA cell line, MSI; HCT 116 CA cell line, MSI. Data are representative of three independent experiments. Error bars represent SEM. *p < 0.05, **p < 0.01, ***p < 0.001.

Based on previous findings suggesting a role for IL-1β in modulating chemotherapeutic response to oxaliplatin in colon cancer cell line HCT 116 (16), we assessed if the presence of IL-1β would interfere with the cellular response to 5-FU, a standard chemotherapeutic agent used in the treatment of CRC. In the literature there are evidence supporting differential response to chemotherapy based on tumor microsatellite status, with studies suggesting that MSI tumors have a decreased response to 5-FU based therapy compared to MSS tumors (3, 20). This might be due to fact that MSI tumors present mutations in genes involved in the Mismatch Repair, which will affect the mechanism of action of 5-FU. Therefore, for this study, we grouped the cell lines as MSS (CHTN-06 and HT-29) and MSI (SB-521 and HCT-116) and evaluated their response to 5-FU treatment. Cells were cultured in complete media, with or without IL-1β, and then exposed to different concentrations of 5-FU (1, 2.5, 5, 10, 15 uM) for 72 hours. Different sensitivities to 5-FU and IC_50_ have been reported in the literature for both CA colon cancer cell lines HT-29 and HCT-116 (21–23), therefore we picked the aforementioned range of concentrations based on these previous findings to test in our new models. As shown in **Figure 3**, IL-1β-treated cells display significantly less sensitivity to 5-FU in all four cell lines. Among all concentrations used, 2.5 and 5 µM represent those for which we saw the largest differences between control and IL-1β treated cells, for either MSS or MSI, or each cell lines. AA CHTN-06 control cells show a mean viability of 55.6% and 30.3% versus a mean viability of 79% and 49.6% when treated with IL-1β, for concentrations of 2.5 and 5 µM of 5-FU respectively. For the same concentrations, HT-29 untreated cells had a mean viability of 58% and 33.3% versus a mean viability of 77.6% and 57% when treated with IL-1β (**Figure 3A**). Interestingly, in our models, both MSI cell lines show an increased sensitivity to 5-FU when compared to MSS cell lines, which is in contrast with previous clinical data reporting decreased sensitivity to 5-FU based therapies in MSI tumors. However, despite being overall more sensitive to the treatment, the presence of IL-1β significantly reduces that sensitivity (mean viability for SB-521: 26.3% vs 46.3% for 2.5 µM, 17% vs 29.6%, for 5 µM – mean viability for HCT-116: 14% vs 38.6 for 2.5µM, 1.3% vs 15.3% for 5 µM) (**Figure 3B**).

**Figure 4:**
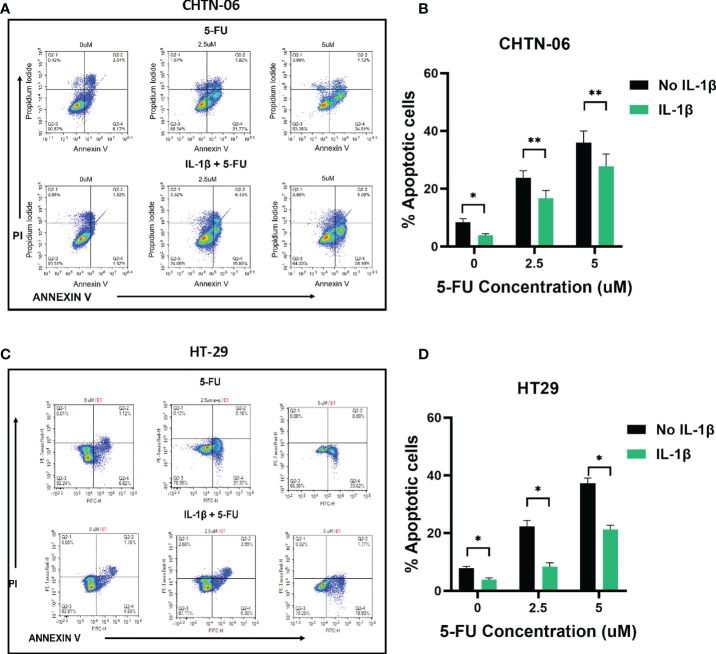
IL-1β decreases 5-FU-induced apoptosis in MSS cell lines. Apoptosis detection by Annexin V/PI staining *via* flow cytometry following treatment with different concentrations of 5-FU (0, 2.5, 5 mM), in the presence or absence of IL-1β. Representative images of flow cytometry data for CHTN-06 **(A)** and HT-29 **(C)**; Percentage of combined early apoptotic (Annexin V+) and late apoptotic (Annexin V+/PI+) cells for CHTN-06 **(B)** and HT-29 **(D)** cell lines. Data are representative of three independent experiments. Error bars represent SEM. *p<0.05, **p<0.01.

**Figure 5:**
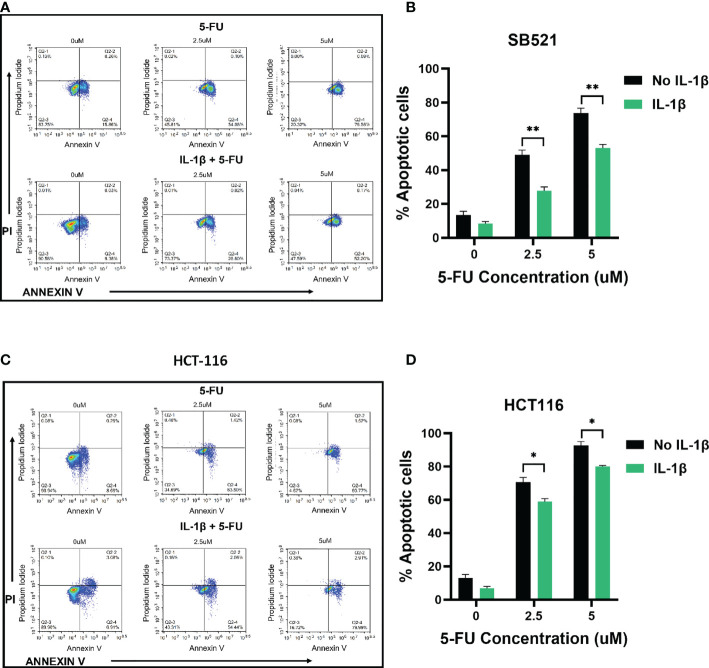
IL-1β decreases 5-FU-induced apoptosis in MSI cell lines. Apoptosis detection by Annexin V/PI staining *via* flow cytometry following treatment with different concentrations of 5-FU (0, 2.5, 5 mM), in the presence or absence of IL-1β. Representative images of flow cytometry data for SB-521 **(A)** and HCT 116 **(C)**; Percentage of combined early apoptotic (Annexin V+) and late apoptotic (Annexin V+/PI +) cells for SB-521 **(B)** and HCT 116 **(D)** cell lines. Data are representative of three independent experiments. Error bars represent SEM. *p<0.05, **p<0.01.

Based on these MTS results, we further assessed whether treating the cells with IL-1β would affect induction of apoptosis by 5-FU. Flow cytometry results demonstrated that treatment with 2.5 and 5 uM 5-FU increases the number of apoptotic (Annexin V+) cells when compared to control untreated cells. In accordance with our previous results, when the cells were pre-treated with 10 ng/ml of IL-1β followed by 5-FU, the number of Annexin V+ cells decreased when compared to 5-FU alone treatment (**Figures 4**, **5**). For CHTN-06, IL-1β decreased apoptotic cells by ~ 30% for both 2.5 and 5 µM of 5-FU; similarly, a ~30% decrease was seen also for HT-29 treated with IL-1β. For MSI cell line SB-521 we saw a decrease in apoptotic cells of ~50% and ~30% for 2.5 and 5 µM of 5-FU respectively, following treatment with IL-1β. For HCT-116, the decrease seen was ~ 15% for both concentrations of 5-FU. Overall, our data suggests that IL-1β might affect the cells sensitivity to 5-FU by decreasing the apoptosis, therefore inducing a consequent increase in cell viability following the chemotherapeutic treatment

### IL-1R/IL-1β axis is responsible for IL-1β induced effects

**Figure 6:**
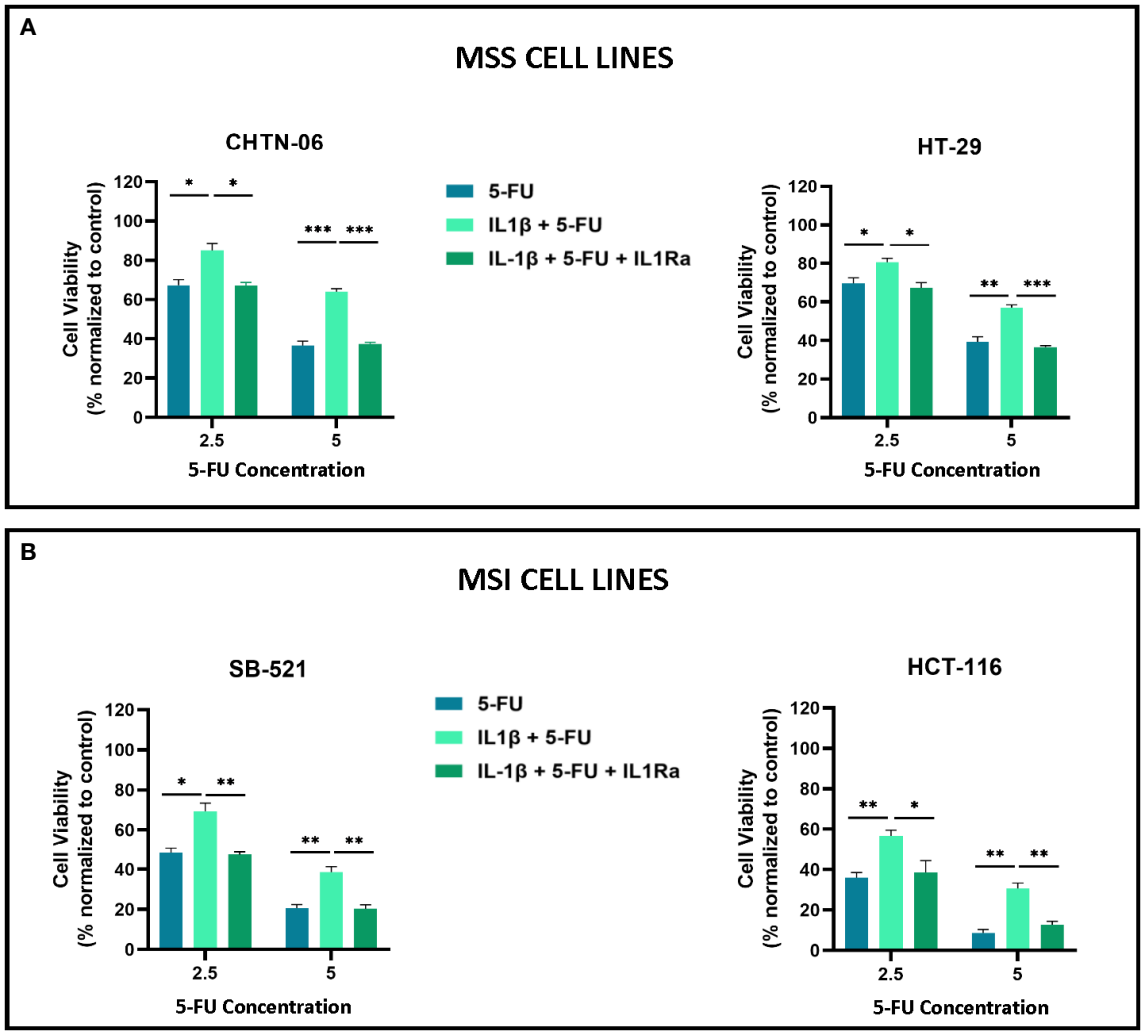
IL-1Ra counteracts IL-1β effects on 5-FU sensitivity. Cell viability following treatment with 5-FU alone, in presence of IL-1β, or in combination with IL-1Ra was determined by MTS assay. Cells were seeded in media, in the presence or absence of 10 ng/ml of IL-1β and then treated with 5-FU alone or in combination with IL-1Ra, and incubated for 72 hours. **(A)** MSS cell lines CHTN-06 and HT-29; **(B)** MSI cell lines SB-521 and HCT-116. Data are representative of three independent experiments. Error bars represent SEM. *p<0.05, **p<0.01, ***p<0.001.

To further investigate IL-1β effects seen on cell proliferation mechanism we used IL-1 Receptor Antagonist (IL-1Ra), which binds non-productively to IL-1R1 with high affinity and prevents IL-1β from initiating the signaling cascade (24). We used an MTS assay to detect changes in cell proliferation when cells were treated with IL-1β alone or in combination with IL-1Ra. We saw that treating the cells with IL-1Ra inhibits the increase in cell proliferation induced by IL-1β, for all the four cell lines. Specifically, at 24 hours of treatment, IL-1β+IL-1Ra treated cells show a significant decrease in cell viability when compared to IL-1β alone treated cells for all the time points (24, 48, 72 hours). Moreover, our data shows that IL-1Ra alone has no significant effect on cell viability when compared to control untreated cells (**Supplementary Figure S2**). In **Figure 3**, we showed how IL-1β reduced the sensitivity to 5-FU treatment, therefore we treated the cell lines with 5-FU and IL-1Ra and evaluated the effects of IL-1β on the combination treatment. The results demonstrate that IL-1Ra reverses the cytokine effects on 5-FU treatment. As shown in **Figure 6**, combination treatment with 5-FU and IL-1Ra (in the presence of IL-1β) significantly reduces cell viability when compared to 5-FU + IL-1β for both 2.5 uM (CHTN-06 p < 0.05, HT-29 p < 0.05, SB-521 p < 0.01, HCT-116 p < 0.05) and 5 uM (CHTN-06 p < 0.001, HT-29 p < 0.001, SB-521 p < 0.01, HCT-116 p < 0.01) of 5-FU.

**Figure 7:**
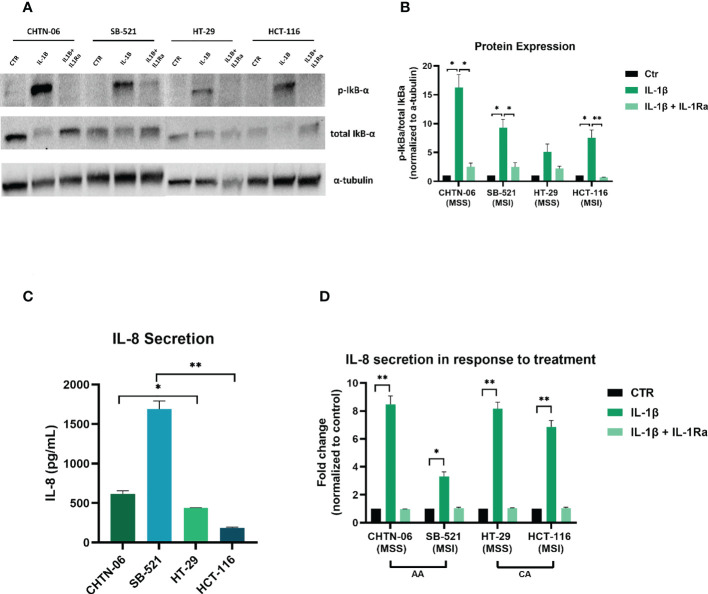
IL-1Ra prevents activation of IL-1β pathway in colon cancer cell lines. Treatment with IL-1Ra prevents phosphorylation of phospho-IkBα and secretion of IL-8. **(A)** Representative images of Western Blot analysis following treatment of cells with either IL-1β alone or in combination with IL-1Ra. **(B)** Graphic visualization of protein expression for phospho-IkBα. Values are expressed as phospho-IkBα/total IkBα ratio, after being normalized to normalized to loading control (α-tubulin). **(C)** Baseline IL-8 secretion in AA and CA colon cancer cell lines. **(D)** Changes in IL-8 secretion following treatment with IL-1β or IL-1β + IL-1Ra Data are representative of three independent experiments. Error bars represent SEM. *p<0.05, **p<0.01.

Our gene expression analysis (**Figure 1**) demonstrated increased expression of genes of the NF-κB pathway in the AA colon cancer cell lines. Therefore, in order to get a better insight into the potential mechanism for the IL-1β-mediated effects, we performed Western Blot analysis to detect activation of the NF-κB pathway, by looking at the expression of phospho-IκBα following treatment with IL-1β. **Figures 7A, B** show that treatment with IL-1β induces a significant increase in phosphorylation for AA cell lines CHTN-06 and SB-521 (p < 0.05) and for CA cell lines HCT-116 (p < 0.05). Our data also demonstrates that IL-1Ra prevents phosphorylation of IκBα protein in all four cell lines; however, the changes seen were significant only for CHTN-06, SB-521 and HCT-116. When comparing the value normalized to control untreated cells, CHTN-06 shows a mean value of 14.9 vs. 2.5 for IL-1β and IL-1Ra treated cells, respectively. Similarly, fold changes are 9.3 vs. 2.5 for SB-521 and 7.5 vs. 0.6 for HCT-116. Overall, we saw a decrease in levels of p-IκBα of 80% for CHTN-06, 70% for SB-521 and 90% for HCT-116 when the cells were treated with IL-1Ra.

After seeing an increase in p-IκBα, which suggests activation of NF-κB following IL-1β treatment, we wanted to evaluate induction of downstream genes of this pathway. We saw in our patient data that tumors from AA present a higher expression of IL8 gene (11), which is a known target of NF-κB. Therefore we performed an ELISA to detect secretion of IL-8 following treatment with IL-1β alone or in combination with IL-1Ra. **Figure 7C** shows the baseline secretion of IL-8 in all four cell lines: interestingly, AA cell lines present a higher secretion of IL-8 even in absence of any treatment, with a 1.4-fold increase for CHTN-06 compared to HT-29, and a 9-fold increase for SB-521 compared to HCT-116. When we treated the cells with IL-1β, we saw a significant increase in the secretion of IL-8 for all four cell lines, with p < 0.01 for CHTN-06, HT-29 and HCT-116, and a p < 0.05 for SB-521. We also saw a significant decrease in IL-8 secretion in IL-1Ra treated cells compared to IL-1β treated cells (CHTN-06: 9-fold, HT-29: 7.7-fold, SB-521: 3.3-fold, HCT-116: 6.6-fold). It is important to note that, for SB-521, we saw the less significant changes following the treatments, and this might be due to the already high secretion of IL-8 in unstimulated cells (**Figure 7D**).

## Discussion

Colorectal cancer has disproportionately affected the AA population, however, there have not been many studies evaluating the causes at the molecular and cellular level, one of the reasons being the absence of *in vitro* models for this specific population. We believe our study represents the first *in vitro* utilization of AA colon cancer cell lines to investigate a role for IL-1β in tumor promotion and response to 5-FU.To our knowledge, our cells lines represent the only *in vitro* colon cancer model derived from AA patients. If taking ATCC as a point of reference, the only colon cell line from an AA individual is CD-18Co. This cell line, however, was isolated from the normal colon tissue of a 2.5-month-old black female displaying a fibroblast-like morphology. As previous studies suggested, regarding inflammatory processes in colon cancer, AA CRC patients appear to be characterized by tumors with higher frequency of *KRAS* mutations and unique mutations in specific genes associated with CRC risk (6, 25), as well as a more pronounced inflammatory response compared to CA patients (7, 8). In Jovov et al. study, the main pathways upregulated in AA CRC patients were those involved in immune-mediated response and inflammation (7). In accordance with this study, we had previously demonstrated that the Cytokine-Cytokine Receptor Interaction pathway was one of the most significantly upregulated in AA patients (11). Moreover, the gene expression analysis found increased levels of the gene encoding for the pro-inflammatory cytokine IL-1β in tumors from AAs compared to CA patients (11). Prior to our findings, Sanabria-Salas MC, et al. had established a correlation between the *IL1B* gene haplotype *IL1B-CGTC*, found in Colombians of African descent, and CRC risk (26). Taken together, these results suggested the need to further investigate inflammatory pathways in the context of racial health disparities, by using more racially diverse models, such as the colon cancer cell lines in the current study.

IL-1β is part of the larger IL-1 family, which includes both agonists (IL-1α, IL-1β, IL-18, IL-33, IL-36) and antagonists (IL-1Ra, IL-36Ra, IL-37, IL-38) (27). IL-1β has been involved in many physiological and pathological processes (28). In relation to cancer, both a pro- and anti-tumorigenic role for IL-1β has been described (28). PCR analysis showed an increased expression of *IL1B* in tumor samples from melanoma, colon and lung cancer patients (29), as did our whole RNA sequencing on tumor samples from both AA and CA patients (11). *In vitro*, IL-1β has been shown to induce cell proliferation and increase invasiveness in colon cancer cell lines (16, 30).. Our results demonstrated that different concentrations of IL-1β elicited distinct responses in AA and CA colon cancer cell lines, with the AA cell lines appearing to be more sensitive to the cytokine stimuli. Based on the goal of our experiments, to ascertain that there would be no issues with the interpretation of our results, we evaluated the secretion levels of both cytokines in cell culture media and we did not detect secretion of either IL-1β or IL-1α from any of the four cell lines (data not shown). These results are not surprising since it has been well documented that IL-1β is most likely derived from the tumor microenvironment (TME) rather than cancer cells. Findings have shown that IL-1β is secreted by immune cells, mainly myeloid cells, which represent an important component of the tumor microenvironment (31). Myeloid cells, also known as Myeloid regulatory cells (MRC) comprise tumor-associated macrophages (TAM), dendritic cells (DC) and myeloid-derived suppressor cells (MDSC). They all contribute to tumor development and progression by decreasing immune surveillance and promoting suppression of anti-tumoral function among immune cells (32).

When we examined the expression of genes that are related to IL-1β activity, we found that there was no difference in the expression of the *IL1R1* gene between the two MSS and the two MSI cell lines. IL-1R1 is part of the IL1R family to which there is interaction with different cytokines in the IL-1 family, initiating the signaling cascade with activation of further downstream genes (33). In CRC patients, higher increased levels of IL-1R1 predicted poor response to Cetuximab (*CTX*) therapy (34). When we evaluated the cell surface protein levels of IL-1R1, we saw that all four cell lines expressed similar levels of the receptor on their membrane. However, when we treated the cells with IL-1β, we saw a more pronounced response in both AA cell lines compared to the CA cell lines. We speculate that the discrepancy in receptor expression and cellular response could be related to differential expression of other members of the IL-1 Receptor family, on which IL-1R1 is dependent on for activation. Following binding to IL-1β, IL-1R1 requires dimerization with IL-1RacP to be activated and initiate the signaling cascade (35). Therefore, in the presence of high expression of IL-1R1, adequate levels of IL-1RacP are needed to transduce IL-1β signaling. Another mechanism that regulates IL-1R1 signaling is IL-1R2 a decoy receptor acting which, due to lack of cytoplasmic domain, binds circulating IL-1β without initiating a signaling cascade (35). If higher levels of IL-1R2 are present on the cell surface, IL-1β will more likely bind to the decoy receptor IL-1R2 than the signaling receptor IL-1R1. Therefore, a decrease in IL-1RacP or an increase in IL-1R2 expression in CA cell lines, might better explain the results we saw in terms of cell proliferation induced by IL-1. Interestingly, we found that AA cell lines SB-521 had increase gene expression of *IL1R2* when compared to HCT-116; however, these findings might not correlate with an effective increase in protein expression in our cell lines.

Our gene expression analysis also shows differential expression for some of the genes of the MAPK pathway, such as *Mitogen-Activated Protein Kinase 3* (*MAPK3*), *Mitogen-Activated Protein Kinase 12* (*MAPK12*), which were upregulated and downregulated, respectively, in the AA colon cancer cell lines. *Mitogen-Activated Protein Kinase 11* (*MAPK11*) and *Mitogen-Activated Protein Kinase 14* (*MAPK14*) were downregulated and upregulated, respectively, in MSI cell lines SB-521. Mitogen-activated protein kinases (MAPKs) are signaling proteins that have been involved in many pathological processes, including inflammation and cancer. Three main MAPK pathways have been identified: the ERK, the JNK and the p38 pathways. JNK and p38 have been both associated with cancer progression and can be activated by a variety of stimuli, and act by regulating the balance between cell survival and cell death and are often dysregulated in cancer cells (36). ERK pathway has been shown to be activated by various stimuli and promote proliferation and invasion in colon cancer (37). Also, p38 has been associated with colon cancer progression as well. For example, inhibition of p38 activation was shown to reduce tumor growth in an *in vivo* model of colon cancer (38), and to increase sensitivity of colon cancer cells to chemotherapeutic agents (39).

We tested the effect of IL-1β on 5-FU treatment in the AA and CA colon cancer cell lines. The mechanism of action of 5-FU is documented and involves inhibition of enzymes required for DNA replication, thereby inhibiting cell replication and proliferation (40). Alteration of genes involved in the mechanism of action of 5-FU have been associated with resistance to the chemotherapeutic agent (40). However, many of the mechanisms of resistance remain unclear. Some recent studies have shown a correlation between cytokine production in patients and response to chemotherapy, suggesting that elevated levels of pro-inflammatory cytokines might be related to lower response to therapy, poor prognosis, and overall decreased survival rates (24, 41, 42). In a study from 2012, Li et al. found that treating CA cell line HCT116 with IL-1β reduced the cellular response to Oxaliplatin, a standard of care chemotherapy for CRC (16). In this study, we linked the presence of IL-1β with a decreased response to 5-FU in both AA and CA cell lines, which is independent of the cell lines microsatellite status. We saw that, presence of IL-1β to the culture media increased the viability and clonogenic potential of the cell lines, as well as decreasing the number of apoptotic cells, counteracting the cytotoxic effects of 5-FU. A possible mechanism for these effects might be related to activation of the NF-κB pathway (43). Our RNAseq analysis identified genes belonging to the NF-κB pathway, specifically *MYD88*, *IRAK3*, *IRAK4* and *TRAF5*, having upregulated expression levels in the two AA cell lines when compared to the CA cell lines. *In vitro* findings have shown that inhibition of NF-κB pathway increases sensitivity to 5-FU in colon cancer cell lines (44, 45). Despite this, we did not see any measurable differences between the AA and CA cell lines in their response to 5-FU. Therefore, we believe more studies are needed to further investigate the mechanisms relating IL-1β and 5-FU response, which might better help explain the different responses to 5-FU seen in patients (4).

Our study investigated a role for IL-1β in AA cell lines and identified possible targets related to the pathway that could offer better therapeutic options for this population. For that reason, we assessed the activity of the IL-1 Receptor Antagonist (IL-1Ra) in these cell line models. IL-1Ra is a naturally occurring cytokine which binds competitively to IL-1 Receptor 1, preventing IL-1 binding and activation of specific inflammatory pathways (33). IL-1Ra has been used as a treatment for rheumatoid arthritis, and more recently IL-1Ra has emerged as a potential anti-cancer therapy (33). It has been shown that IL-1Ra is involved in suppression of carcinogenesis, metastasis and inhibition of oncogenic pathways in many types of cancer, including CRC (33). Clinical studies have determined an association between gene polymorphisms and circulating levels of IL-1Ra, and a good prognostic value. Specifically, it has been shown that CRC patients carrying the T/T allele, which is associated with increased levels of circulating IL-1Ra, have a higher survival rate compared to those carrying the C/C allele (46). Interestingly, our data shows an increased expression for *IL1RN* (IL-1Ra) in one of the AA cell lines. Despite this finding, treatment with IL-1β still significantly increased cell proliferation in this cell line. Therefore, when we tested the secretion levels of IL-1Ra we saw no secretion of the cytokine in any of the cell lines, in the presence or absence of IL-1β treatment. As shown in a recent study (47), our results also suggest a role for IL-1Ra in preventing IL-1β-mediated effects on 5-FU response.

In addition, our results demonstrated that the increase of both phospho-IκBα expression and IL-8 secretion seen following treatment with IL-1β, was prevented when the cells were pre-treated with IL-1Ra. Our findings are in accordance with previous studies showing a role for IL-1Ra in blocking both IL-1α and IL-1β-mediated angiogenesis, *via* NF-κB pathway (48, 49). NF-κB regulates transcription of multiple genes involved in critical cellular mechanisms, therefore, in normal cells, NF-κB activation is strictly regulated and signaling is rapidly turned off. In cancer cells however, NF-κB pathway is often dysregulated so that cells could evade apoptosis and proliferate uncontrollably. In the context of colorectal cancer, NF-κB and some of its regulated genes, have been found to be expressed at higher levels in tumors samples, and appear to correlate with a worse prognosis (50, 51). It has been shown that NF-kB induces transcription of many genes, including pro-inflammatory cytokines, such as IL-8, IL-6 and TNF-α (44). Our findings show an increased baseline secretion of IL-8 in AA cell lines which is further increased following treatment with IL-1β. Secretion of IL-8 from cancer cells into the tumor microenvironment is associated with an increased recruitment and accumulation of myeloid suppressor cells at the tumor site and it correlates with poor outcomes (52). Interestingly, we have previously showed an increase in both *IL1B* and *IL8* gene expression in AA tumor samples, increased presence of myeloid cells, as well as upregulation of genes related to myeloid-derived suppressor cells (11). In light of all these findings, we suggest a role for IL-1β, in which higher expression and secretion of this cytokine induces activation of pro-inflammatory pathways and contributes to tumor progression

In the present study, we assessed for the first time, the response to the pro-inflammatory cytokine IL-1β in AA colon cancer *in vitro* models, with the purpose of better understanding the mechanisms behind the lower response to treatment as well as overall poor prognosis in AA patients (4). We demonstrated a pro-tumorigenic role for IL-1β and the ability for the cytokine to interfere with the effects of the chemotherapeutic agent 5-FU. We observed a more pronounced response to IL-1β in terms of cell proliferation and differential expression of genes and pro-inflammatory proteins between AA and CA cell lines, which might suggest that specific cellular functions might be regulated *via* different pathways in these cell lines. We also demonstrated a higher baseline expression of pro-inflammatory genes and cytokine IL-8 secretion in our AA cell lines, suggesting an important role for inflammatory pathways to be further investigated, in the context of cancer health disparities. Overall, our data demonstrated differential expression of pro-inflammatory genes and distinct responses to inflammatory stimuli between AA and CA colon cancer cell lines. However, more work is needed to better address the issue of cancer racial disparities and to better serve the cancer necessities of underrepresented communities. We believe our *in vitro* models represent the first step towards identifying new molecules that differ between the AA and CA populations, and that could represent important biomarkers for diagnosis, response to treatment and new therapeutic strategies.

## Data availability statement

The original contributions presented in the study are included in the article/**Supplementary Material**. Further inquiries can be directed to the corresponding authors.

## Author contributions

MS, JP, LM and JW contributed to conception and design of the study. MS, JP, TR and JG performed the research (data acquisition). MS, JP, JZ and LM performed analysis and interpretation of data. LM supervised the research. MS wrote the draft of the manuscript. All authors contributed to manuscript revision, read and approved the submitted version.

## Funding

Funding was also provided by the Stony Brook University Cancer Center, 1P20CA192994-01A1

## Acknowledgments

The authors wish to thank Stanley Soroka and Sofia Tortora-Morel for their edits, Dr. Christopher Roman for his knowledge and for his constant support.

## Conflict of interest

The authors declare that the research was conducted in the absence of any commercial or financial relationships that could be construed as a potential conflict of interest.

## Publisher’s note

All claims expressed in this article are solely those of the authors and do not necessarily represent those of their affiliated organizations, or those of the publisher, the editors and the reviewers. Any product that may be evaluated in this article, or claim that may be made by its manufacturer, is not guaranteed or endorsed by the publisher.

## Supplementary material

Supplementary Figure 1 IL-1β induces proliferation in AA and CA colon cancer cell lines. Changes in cell proliferation were detected *via* MTS Assay in both AA and CA colon cancer cell lines. Cells were seeded in media containing increasing concentrations of IL-1β (1, 5, 10, 30, 60 ng/ml) and changes in cell viability were detected after 96 hours. MSS cell lines (CHTN-96 and HT-29) **(A)**, MSI cell lines (SB-521 and HCT-116) **(B)**. Data representative of three independent experiments. Error bars represent SEM. *p < 0.05, **p < 0.01, ***p < 0.001.

Supplementary Figure 2 IL-1Ra inhibits IL-1β-induced proliferation in colon cancer cell lines. Cells were seeded in media without (Control and IL-1Ra) or with (IL-1β and IL-1β + IL-1Ra) 10 ng/ml of IL-1β and incubated for 24 hours. The following day IL-1Ra (10 ug/ml) was added and treatment was carried out for 24 hours **(A)**, 48 hours **(B)** and 72 hours **(C)**. Cell viability was detected *via* MTS assay. Data are representative of three independent experiments. Error bars represent SEM. *p<0.05, **p<0.01, ***p<0.001.

## References

[1] American Cancer Society. Cancer facts & figures 2021. Atlanta: American Cancer Society (2021).

[2] Popat S Hubner R Houlston RS. Systematic review of microsatellite instability and colorectal cancer prognosis. J Clin Oncol (2005) 23(3):609–18. doi: 10.1200/JCO.2005.01.086 15659508

[3] Carethers JM Smith EJ Behling CA Nguyen L Tajima A Doctolero RT. Use of 5-fluorouracil and survival in patients with microsatellite-unstable colorectal cancer. Gastroenterol (2004) 126(2):394–401. doi: 10.1053/j.gastro.2003.12.023 14762775

[4] Dimou A Syrigos KN Saif MW. Disparities in colorectal cancer in African-americans vs whites: before and after diagnosis. World J Gastroenterol (2009) 15(30):3734–43. doi: 10.3748/wjg.15.3734 PMC2726450 19673013

[5] Carethers JM Murali B Yang B Doctolero RT Tajima A Basa R. Influence of race on microsatellite instability and CD8+ T cell infiltration in colon cancer. PloS One (2014) 9(6):e100461. doi: 10.1371/journal.pone.0100461 24956473 PMC4067325

[6] Guda K Veigl ML Varadan V Nosrati A Ravi L Lutterbaugh J. Novel recurrently mutated genes in African American colon cancers. Proc Natl Acad Sci U S A (2015) 112(4):1149–54. doi: 10.1073/pnas.1417064112 PMC4313860 25583493

[7] Jovov B Araujo-Perez F Sigel CS Stratford JK McCoy AN Yeh JJ. Differential gene expression between African American and European American colorectal cancer patients. PloS One (2012) 7(1):e30168. doi: 10.1371/journal.pone.0030168 22276153 PMC3261881

[8] Wang X Ji P Zhang Y LaComb JF Tian X Li E. Aberrant DNA methylation: Implications in racial health disparity. PloS One (2016) 11(4):e0153125. doi: 10.1371/journal.pone.0153125 27111221 PMC4844165

[9] American Cancer Society. Cancer facts & figures for African American/Black people 2022-2024. Atlanta: American Cancer Society (2022).

[10] Le DT Uram JN Wang H Bartlett BR Kemberling H Eyring AD. PD-1 blockade in tumors with mismatch-repair deficiency. N Engl J Med (2015) 372(26):2509–20. doi: 10.1056/NEJMoa1500596 PMC4481136 26028255

[11] Paredes J Zabaleta J Garai J Ji P Imtiaz S Spagnardi M. Immune-related gene expression and cytokine secretion is reduced among African American colon cancer patients. Front Oncol (2020) 10:1498. doi: 10.3389/fonc.2020.01498 32983990 PMC7492388

[12] Klampfer L. Cytokines, inflammation and colon cancer. Curr Cancer Drug Targets (2011) 11(4):451–64. doi: 10.2174/156800911795538066 PMC3540985 21247378

[13] Krzystek-Korpacka M Diakowska D Kapturkiewicz B Bębenek M Gamian A. Profiles of circulating inflammatory cytokines in colorectal cancer (CRC), high cancer risk conditions, and health are distinct. possible implications for CRC screening and surveillance. Cancer Lett (2013) 337(1):107–14. doi: 10.1016/j.canlet.2013.05.033 23726839

[14] Terzić J Grivennikov S Karin E Karin M. Inflammation and colon cancer. Gastroenterology (2010) 138(6):2101–2114.e5. doi: 10.1053/j.gastro.2010.01.058 20420949

[15] Kaler P Godasi BN Augenlicht L Klampfer L. The NF-κB/AKT-dependent induction of wnt signaling in colon cancer cells by macrophages and IL-1β. Cancer Microenviron (2009) 2(1):69–80. doi: 10.1007/s12307-009-0030-y 19779850 PMC2787930

[16] Li Y Wang L Pappan L Galliher-Beckley A Shi J. IL-1β promotes stemness and invasiveness of colon cancer cells through Zeb1 activation. Mol Cancer (2012) 11:87. doi: 10.1186/1476-4598-11-87 23174018 PMC3532073

[17] Paredes J Ji P Lacomb JF Shroyer KR Martello LA Williams JL. Establishment of three novel cell lines derived from African American patients with colorectal carcinoma: A unique tool for assessing racial health disparity. Int J Oncol (2018) 53(4):1516–28. doi: 10.3892/ijo.2018.4510 PMC6086619 30066857

[18] Guo R Qin Y Shi P Xie J Chou M Chen Y. IL-1β promotes proliferation and migration of gallbladder cancer cells *via* twist activation. Oncol Lett (2016) 12(6):4749–55. doi: 10.3892/ol.2016.5254 PMC5228334 28105184

[19] Johnstone M Bennett N Standifer C Smith A Han A Bettaieb A. Characterization of the pro-inflammatory cytokine IL-1β on butyrate oxidation in colorectal cancer cells. J Cell Biochem (2017) 118(6):1614–21. doi: 10.1002/jcb.25824 27922186

[20] Jo WS Carethers JM. Chemotherapeutic implications in microsatellite unstable colorectal cancer. Cancer biomark (2006) 2(1-2):51–60. doi: 10.3233/cbm-2006-21-206 17192059 PMC4948976

[21] Bracht K Nicholls AM Liu Y Bodmer WF. 5-fluorouracil response in a large panel of colorectal cancer cell lines is associated with mismatch repair deficiency. Br J Cancer (2010) 103(3):340–6. doi: 10.1038/sj.bjc.6605780 PMC2920028 20606684

[22] Liu Z Yu M Fei B Sun J Wang D. Nonhomologous end joining key factor XLF enhances both 5-florouracil and oxaliplatin resistance in colorectal cancer. Onco Targets Ther (2019) 12:2095–104. doi: 10.2147/OTT.S192923 PMC6430989 30936724

[23] Ishikawa K Kawano Y Arihara Y Kubo T Takada K Murase K. BH3 profiling discriminates the anti-apoptotic status of 5-fluorouracil-resistant colon cancer cells. Oncol Rep (2019) 42(6):2416–25. doi: 10.3892/or.2019.7373 PMC6826312 31638265

[24] Kim JW Koh Y Kim DW Ahn YO Kim TM Han SW. Clinical implications of VEGF, TGF-β1, and IL-1β in patients with advanced non-small cell lung cancer. Cancer Res Treat (2013) 45(4):325–33. doi: 10.4143/crt.2013.45.4.325 PMC3893330 24454005

[25] Yoon HH Shi Q Alberts SR Goldberg RM Thibodeau SN Sargent DJ. Alliance for clinical trials in oncology. racial differences in BRAF/KRAS mutation rates and survival in stage III colon cancer patients. J Natl Cancer Inst (2015) 107(10):djv186. doi: 10.1093/jnci/djv186 26160882 PMC5758035

[26] Sanabria-Salas MC Hernández-Suárez G Umaña-Pérez A Rawlik K Tenesa A Serrano-López ML. IL1B-CGTC haplotype is associated with colorectal cancer in admixed individuals with increased African ancestry. Sci Rep (2017) 7:41920. doi: 10.1038/srep41920 28157220 PMC5291207

[27] Dinarello CA. Overview of the IL-1 family in innate inflammation and acquired immunity. Immunol Rev (2018) 281(1):8–27. doi: 10.1111/imr.12621 29247995 PMC5756628

[28] Baker KJ Houston A Brint E. IL-1 family members in cancer; two sides to every story. Front Immunol (2019) 10:1197. doi: 10.3389/fimmu.2019.01197 31231372 PMC6567883

[29] Elaraj DM Weinreich DM Varghese S Puhlmann M Hewitt SM Carroll NM. The role of interleukin 1 in growth and metastasis of human cancer xenografts. Clin Cancer Res (2006) 12(4):1088–96. doi: 10.1158/1078-0432.CCR-05-1603 16489061

[30] Hai Ping P Feng Bo T Li L Nan Hui Y Hong Z. IL-1β/NF-kb signaling promotes colorectal cancer cell growth through miR-181a/PTEN axis. Arch Biochem Biophys (2016) 604:20–6. doi: 10.1016/j.abb.2016.06.001 27264420

[31] Litmanovich A Khazim K Cohen I. The role of interleukin-1 in the pathogenesis of cancer and its potential as a therapeutic target in clinical practice. Oncol Ther (2018) 6(2):109–27. doi: 10.1007/s40487-018-0089-z PMC7359982 32700032

[32] Sieminska I Baran J. Myeloid-derived suppressor cells in colorectal cancer. Front Immunol (2020) 11:1526. doi: 10.3389/fimmu.2020.01526 32849517 PMC7426395

[33] Lewis AM Varghese S Xu H Alexander HR. Interleukin-1 and cancer progression: the emerging role of interleukin-1 receptor antagonist as a novel therapeutic agent in cancer treatment. J Transl Med (2006) 4:48. doi: 10.1186/1479-5876-4-48 17096856 PMC1660548

[34] Gelfo V Mazzeschi M Grilli G Lindzen M Santi S D'Uva G. A novel role for the interleukin-1 receptor axis in resistance to anti-EGFR therapy. Cancers (Basel) (2018) 10(10):355. doi: 10.3390/cancers10100355 30261609 PMC6210663

[35] Boraschi D Italiani P Weil S Martin MU. The family of the interleukin-1 receptors. Immunol Rev (2018) 281(1):197–232. doi: 10.1111/imr.12606 29248002

[36] Braicu C Buse M Busuioc C Drula R Gulei D Raduly L. A comprehensive review on MAPK: A promising therapeutic target in cancer. Cancers (Basel) (2019) 11(10):1618. doi: 10.3390/cancers11101618 31652660 PMC6827047

[37] Zhou G Yang J Song P. Correlation of ERK/MAPK signaling pathway with proliferation and apoptosis of colon cancer cells. Oncol Lett (2019) 17(2):2266–70. doi: 10.3892/ol.2018.9857 PMC6341783 30675292

[38] Peng L Xing X Li W Qu L Meng L Lian S. PRL-3 promotes the motility, invasion, and metastasis of LoVo colon cancer cells through PRL-3-integrin beta1-ERK1/2 and-MMP2 signaling. Mol Cancer (2009) 8:110. doi: 10.1186/1476-4598-8-110 19930715 PMC2792223

[39] Gupta J Igea A Papaioannou M Lopez-Casas PP Llonch E Hidalgo M. Pharmacological inhibition of p38 MAPK reduces tumor growth in patient-derived xenografts from colon tumors. Oncotarget (2015) 6(11):8539–51. doi: 10.18632/oncotarget.3816 PMC4496165 25890501

[40] Zhang N Yin Y Xu SJ Chen WS. 5-fluorouracil: mechanisms of resistance and reversal strategies. Molecules (2008) 13(8):1551–69. doi: 10.3390/molecules13081551 PMC6244944 18794772

[41] Mitsunaga S Ikeda M Shimizu S Ohno I Furuse J Inagaki M. Serum levels of IL-6 and IL-1β can predict the efficacy of gemcitabine in patients with advanced pancreatic cancer. Br J Cancer (2013) 108(10):2063–9. doi: 10.1038/bjc.2013.174 PMC3670479 23591198

[42] Hu F Song D Yan Y Huang C Shen C Lan J. IL-6 regulates autophagy and chemotherapy resistance by promoting BECN1 phosphorylation. Nat Commun (2021) 12(1):3651. doi: 10.1038/s41467-021-23923-1 34131122 PMC8206314

[43] Ishida K Nishizuka SS Chiba T Ikeda M Kume K Endo F. Molecular marker identification for relapse prediction in 5-FU-based adjuvant chemotherapy in gastric and colorectal cancers. PloS One (2012) 7(8):e43236. doi: 10.1371/journal.pone.0043236 22905237 PMC3419205

[44] Voboril R Hochwald SN Li J Brank A Weberova J Wessels F. Inhibition of NF-kappa b augments sensitivity to 5-fluorouracil/folinic acid in colon cancer. J Surg Res (2004) 120(2):178–88. doi: 10.1016/j.jss.2003.11.023 15234211

[45] Cai BQ Chen WM Zhao J Hou W Tang JC. Nrf3 promotes 5-FU resistance in colorectal cancer cells *via* the NF-κB/BCL-2 signaling pathway *In vitro* and in vivo. J Oncol (2021) 2021:9355555. doi: 10.1155/2021/9355555 34795760 PMC8595022

[46] Graziano F Ruzzo A Canestrari E Loupakis F Santini D Rulli E. Variations in the interleukin-1 receptor antagonist gene impact on survival of patients with advanced colorectal cancer. Pharmacogenomics J (2009) 9(1):78–84. doi: 10.1038/tpj.2008.16 19104506

[47] Yan Y Lin HW Zhuang ZN Li M Guo S. Interleukin-1 receptor antagonist enhances chemosensitivity to fluorouracil in treatment of kras mutant colon cancer. World J Gastrointest Oncol (2020) 12(8):877–92. doi: 10.4251/wjgo.v12.i8.877 PMC7443842 32879665

[48] Taniguchi K Karin M. NF-κB, inflammation, immunity and cancer: coming of age. Nat Rev Immunol (2018) 18(5):309–24. doi: 10.1038/nri.2017.142 29379212

[49] Xia Y Shen S Verma IM. NF-κB, an active player in human cancers. Cancer Immunol Res (2014) 2(9):823–30. doi: 10.1158/2326-6066.CIR-14-0112 PMC4155602 25187272

[50] Negi RR Rana SV Gupta R Gupta V Chadha VD Dhawan DK. Increased nuclear factor-κB/RelA expression levels in human colorectal carcinoma in north Indian patients. Indian J Clin Biochem (2018) 33(4):473–8. doi: 10.1007/s12291-017-0703-0 PMC6170227 30319196

[51] González-Quezada BA Santana-Bejarano UF Corona-Rivera A Pimentel-Gutiérrez HJ Silva-Cruz R Ortega-De-la-Torre C. Expression profile of NF-κB regulated genes in sporadic colorectal cancer patients. Oncol Lett (2018) 15(5):7344–54. doi: 10.3892/ol.2018.8201 PMC5962836 29849793

[52] Tobin RP Jordan KR Kapoor P Spongberg E Davis D Vorwald VM. IL-6 and IL-8 are linked with myeloid-derived suppressor cell accumulation and correlate with poor clinical outcomes in melanoma patients. Front Oncol (2019) 9:1223. doi: 10.3389/fonc.2019.01223 31781510 PMC6857649

