# Supplementary figures and images for: IL-1β enhances cell viability and decreases 5-FU sensitivity in novel colon cancer cell lines derived from African American patients
Source: Front Oncol. 2022 Dec 1;12:1010380. doi: 10.3389/fonc.2022.1010380 (PMC9754664; doi:10.3389/fonc.2022.1010380)

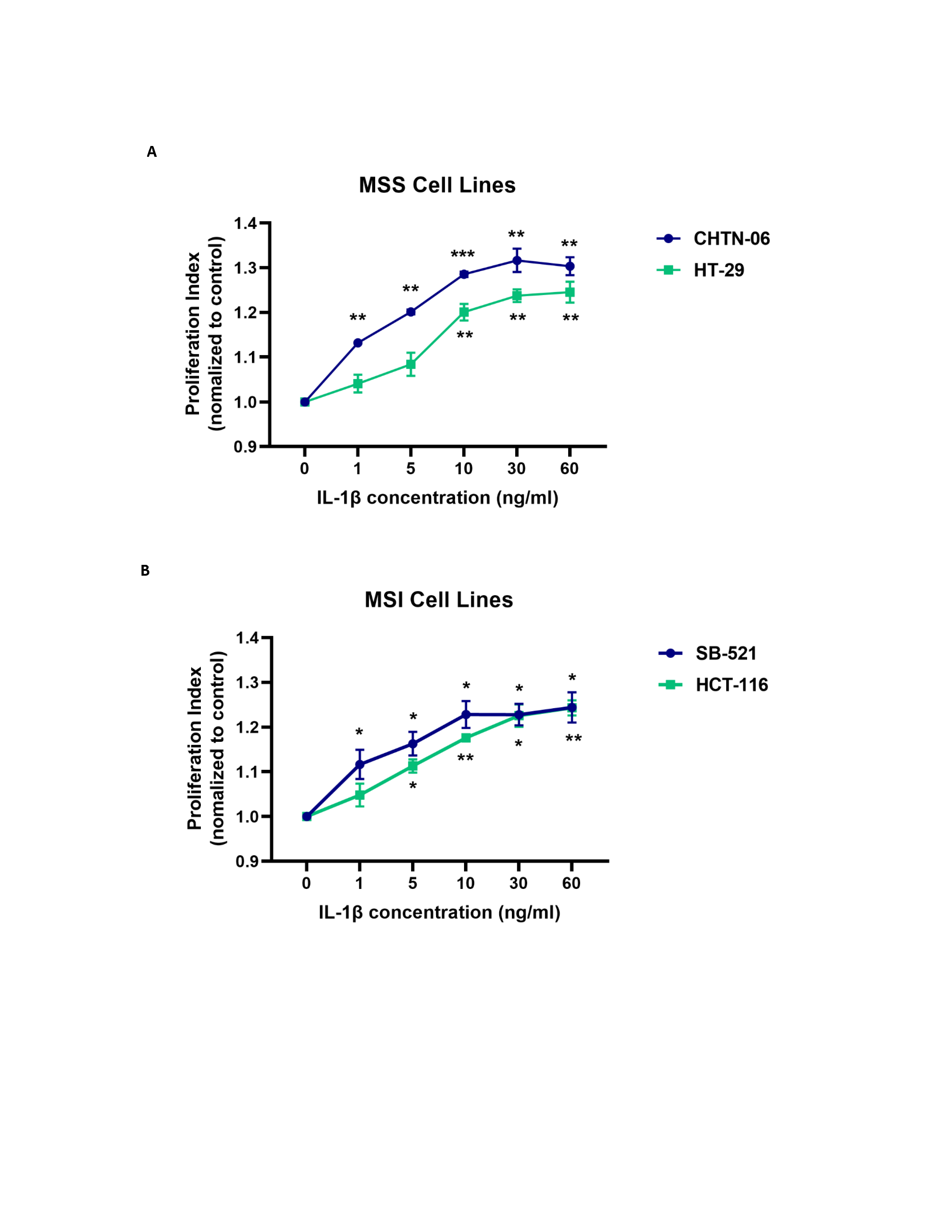

Supplement: Supplementary Figure 1 — IL-1β induces proliferation in AA and CA colon cancer cell lines. Changes in cell proliferation were detected via MTS Assay in both AA and CA colon cancer cell lines. Cells were seeded in media containing increasing concentrations of IL-1β (1, 5, 10, 30, 60 ng/ml) and changes in cell viability were detected after 96 hours. MSS cell lines (CHTN-96 and HT-29) (A), MSI cell lines (SB-521 and HCT-116) (B). Data representative of three independent experiments. Error bars represent SEM. *p < 0.05, **p < 0.01, ***p < 0.001. [file Image_1.tif]

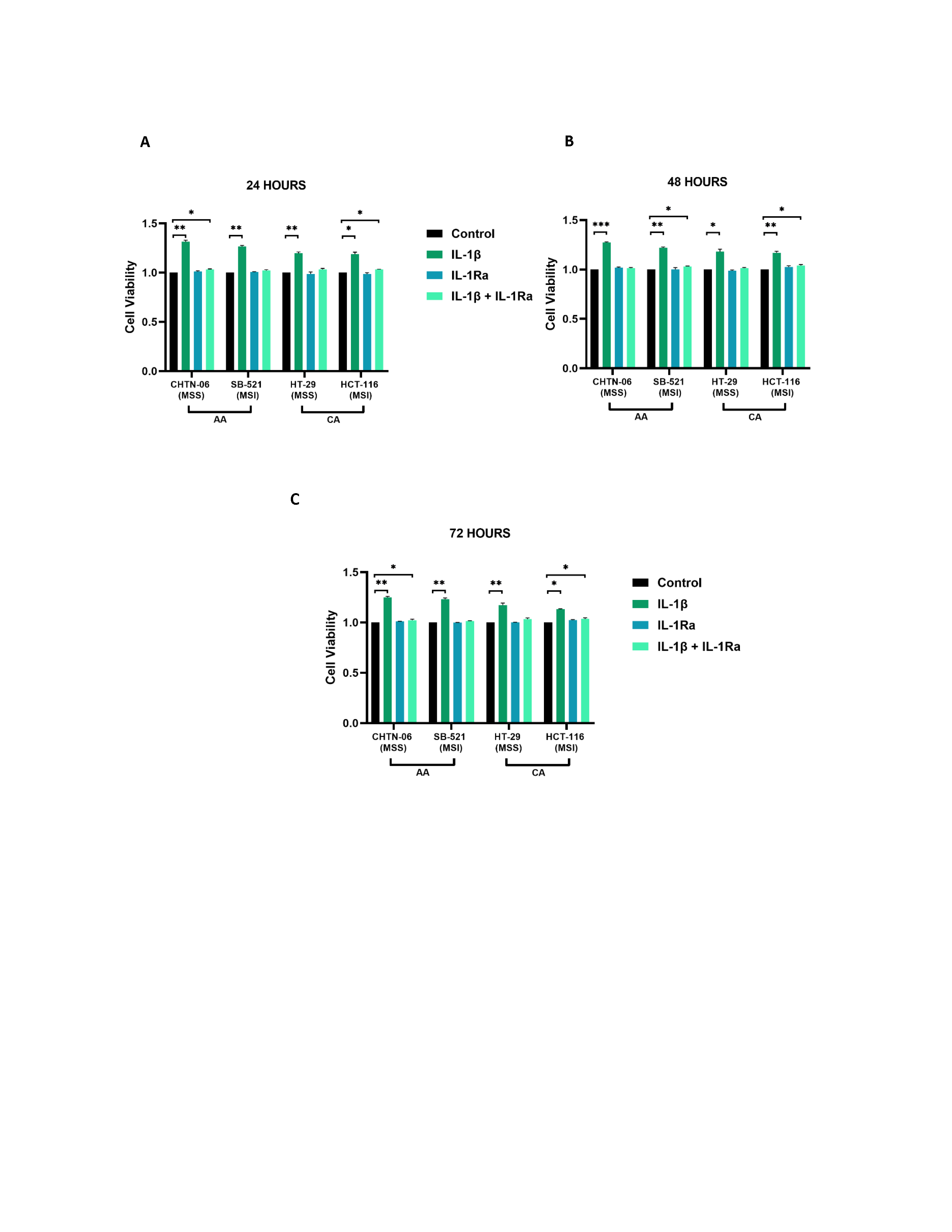

Supplement: Supplementary Figure 2 — IL-1Ra inhibits IL-1β-induced proliferation in colon cancer cell lines. Cells were seeded in media without (Control and IL-1Ra) or with (IL-1β and IL-1β + IL-1Ra) 10 ng/ml of IL-1β and incubated for 24 hours. The following day IL-1Ra (10 ug/ml) was added and treatment was carried out for 24 hours (A), 48 hours (B) and 72 hours (C). Cell viability was detected via MTS assay. Data are representative of three independent experiments. Error bars represent SEM. *p<0.05, **p<0.01, ***p<0.001. [file Image_2.tif]
